# Gut Microbiota Alterations and Intestinal Pathology in Healthy, Moribund, and Recently Dead Mastacembelus Armatus from a Pond Aquaculture System

**DOI:** 10.3390/ani16213350

**Published:** 2026-10-23

**Authors:** Jun Yu, Pengfei Li, Qiwei Guo, Qing Li, Chang Xu, Ngoc Tuan Tran, Xiuli Chen, Wei Yang, Junjun Sun, Shengkang Li, Ming Zhang

**Affiliations:** 1 Department of Cuisine and Nutrition, Hanshan Normal University, Chaozhou 521041, China; 2 Guangdong Provincial Key Laboratory of Marine Biology, Shantou University, Shantou 515063, China; 3 Institute of Marine Sciences, Shantou University, Shantou 515063, China; 4 Guangxi Key Laboratory of Aquatic Genetic Breeding and Healthy Aquaculture, Guangxi Academy of Fishery Sciences, Nanning 530021, China; 5 Key Laboratory of Aquatic Functional Feed and Environmental Regulation of Fujian Province, Fujian DBN Aquatic Sci. & Tech. Co., Ltd., Zhangzhou 363500, China

**Keywords:** *Mastacembelus armatus*, gut microbiota, dysbiosis, sediment microbiome, source tracking

## Abstract

Spiny eel (*Mastacembelus armatus*) pond aquaculture suffers from recurrent nonspecific mortality, yet the associations between gut-microbiota shifts, host histological alterations, and fish physiological status, as well as the potential roles of sediment microbiota, remain unclear. In this study, we collected healthy (H), moribund (M), and recently dead (D) fish, as well as pond sediment (S), and performed histopathological examination and 16S rRNA amplicon sequencing combined with FEAST source-tracking analysis. Histology revealed severe intestinal villus atrophy, epithelial shedding, and extensive hepatic vacuolation in M fish. Results of 16S rRNA sequencing revealed a significant decrease in the alpha diversity (Chao1) of gut microbial richness in M and D groups, compared with healthy (H) (*p* < 0.05). Healthy guts were dominated by Proteobacteria and the keystone genus *Cetobacterium* (about 40% relative abundance). In M/D, *Cetobacterium* nearly disappeared, while Firmicutes and Fusobacteriota became predominant, with potentially opportunistic taxa (Fusobacterium, Clostridiaceae) showing markedly higher relative abundance. Beta-diversity and LEfSe analyses showed overlapping microbial communities between M and D fish, which were distinct from those of healthy (H) fish. FEAST modeling output showed that the conditionally estimated sediment-derived proportion increased from approximately 28% in healthy (H) to ~99% in M fish. Functional profiles predicted by PICRUSt2 shifted from oxidative phosphorylation toward peptidoglycan biosynthesis and peptidase-related functional potentials. Collectively, our results demonstrate strong associations between gut-microbiota disruption, intestinal barrier impairment, and fish physiological decline. We observed associations between damaged intestinal barrier status and increased sediment-attributed fractions of potentially opportunistic microbes within the gut microbiota.

## Simple Summary

Spiny eel farming in ponds often suffers from repeated deaths for unknown reasons. Farmers need better ways to predict and prevent these losses. We studied healthy eels, moribund eels, recently dead eels, and the sediment at the bottom of their ponds. We examined their body tissues and the communities of bacteria living in their guts. We complemented our analyses with FEAST conditional source-tracking estimation. The results showed that moribund eels had serious damage to the gut and liver. The variety of gut bacteria dropped sharply. In healthy eels, helpful bacteria were common, but in moribund and dead eels these helpful bacteria almost disappeared, and potentially opportunistic bacterial groups increased greatly. The bacterial communities in moribund and recently dead eels were similar to each other but very different from those in healthy eels. As the fish became ill, FEAST conditional source-tracking estimated that the proportion of sediment-associated bacterial signals rose from about one quarter to almost all of the community. These observed associations indicate profound gut microbiota disruption is coupled with host physiological decline. When the gut’s protective lining breaks down, pond sediment may help potentially opportunistic bacterial groups grow. This work can help farmers monitor pond conditions and fish health, reduce unexplained deaths, and support more sustainable eel farming.

## 1. Introduction

The spiny eel (*Mastacembelus armatus*) is a carnivorous demersal freshwater fish belonging to the family *Mastacembelidae* (Synbranchiformes), widely distributed across South and Southeast Asia and the southern watersheds of China [1,2,3]. Owing to its tender flesh and high nutritional value, this species commands considerable economic importance in Asian aquaculture markets, with expanding market demand and increasing pond culture production across major Asian producing regions [4]. In recent years, successive breakthroughs in artificial propagation and larval rearing techniques have spurred the rapid expansion of intensive pond culture, positioning the spiny eel as a high-value commodity for regional aquaculture diversification [5]. However, the intensification of farming has resulted in the continuous deposition of uneaten feed and feces, which alter sediment microbial community structure and exacerbate the accumulation of anoxic conditions and toxic metabolites such as ammonia and nitrite [6,7]. As an obligate benthic species, the spiny eel remains in intimate contact with the sediment throughout its life cycle and is disproportionately sensitive to fluctuations in benthic habitat quality compared to pelagic fish [8]. It has been hypothesized that chronic exposure to degraded sediments induces persistent physiological stress, intestinal barrier disruption, and immunosuppression, which may ultimately contribute to progressive physiological decline and nonspecific mortality [9,10].

The gut microbiota of teleost fishes is a core regulatory system underpinning mucosal immune homeostasis, and it is profoundly involved in nutrient metabolism, maintenance of the intestinal epithelial barrier, and innate immune responses. A structurally stable gut microbial community is therefore a prerequisite for host health [11,12,13]. In healthy spiny eel, the gut harbors a stable symbiotic consortium dominated by Proteobacteria and Fusobacteriota, with *Cetobacterium* serving as a keystone commensal that contributes to host homeostasis through vitamin synthesis, fortification of the epithelial barrier, and modulation of mucosal immunity [14,15]. Under environmental stressors such as elevated ammonia or nitrite, this microbial equilibrium is disrupted, with beneficial symbionts markedly declining while potentially opportunistic taxa such as *Aeromonas veronii* and members of Clostridiaceae proliferate aberrantly. The resulting dysbiosis further compromises intestinal barrier integrity, triggers local inflammation and systemic immunosuppression, and sets in motion a vicious cycle whereby gut–immune crosstalk accelerates host physiological collapse [16,17,18,19,20].

Aquaculture pond sediment represents the most species-rich microbial reservoir in the pond ecosystem, harboring diverse functional guilds and potentially opportunistic taxa, and its community structure is a key indicator of environmental health [21,22,23]. For demersal fish, there is a continuous bidirectional exchange of microorganisms between the gut and the sediment [24,25,26]. Sediment microbes can colonize the host gut via ingestion and surface contact, thereby participating in the assembly of the gut microbiota. Moribund individuals and decomposing carcasses release endogenous gut bacteria and tissue-derived organic matter back into the surrounding sediment, altering the structure of the benthic community, elevating loads of potentially opportunistic taxa in the environment, and creating a positive-feedback loop for pathogen propagation [26,27]. Nevertheless, most studies to date have examined the gut and sediment microbiota in isolation. Consequently, the synergistic dynamics between host physiological decline and sediment microbial communities have not been fully explored. In particular, targeted investigations of the sediment microecological characteristics surrounding dead individuals are scarce, and the coupled interaction mechanisms between host and environmental microbiota in benthic aquaculture systems remain to be systematically elucidated [28,29].

Current research on spiny eel has primarily focused on genetic improvement [3,30,31], artificial propagation and embryonic development [9,32], and efficient pond culture techniques [5]. Stress-triggered gut dysbiosis is tightly coupled with intestinal tissue damage and increases host mortality risk in freshwater fish [33,34]. As an important microbial reservoir in ponds, sediment microbiota correlates with fish health and exchanges microbes with benthic fish guts [35]. Furthermore, gut microbial signatures show great potential as non-invasive health biomarkers for farmed fish [12,36]. Nevertheless, few studies have profiled gut microbiota shifts across healthy, moribund, and dead stages of pond-reared spiny eels, nor explored integrated associations among gut dysbiosis, intestinal lesions, and sediment-derived microbial profiles.

To fill this gap, we characterized gut-microbiota dynamics and intestinal pathological alterations during natural physiological decline of pond-cultured spiny eels, and assessed associations between sediment microbiota and gut microbial shifts to screen candidate microbial markers for the moribund status. We compared gut microbiota and intestinal histology of healthy, moribund, and dead individuals from one pond against corresponding sediment samples. This work describes the progressive pattern of gut dysbiosis and tissue injury along the mortality gradient and provides preliminary references for spiny eel aquaculture health management.

## 2. Materials and Methods

### 2.1. Experimental Designs and Sampling

The spiny eels (body weight about 20–35 g, body length about 11.0–15.0 cm) were obtained from a nursery pond located in the Han River basin (Chaozhou, China). Individuals were assigned to three groups (*n* = 6 per group) based on their physiological status: (1) healthy group (H), consisting of fish with normal swimming behavior, intact body surface, abundant mucus, and active stress responses; (2) near-death or moribund group (M), comprising fish showing loss of equilibrium, weak stress responses, and gross pathological signs such as skin ulceration and hepatic cyanosis; and (3) recently dead group (D), consisting of freshly dead individuals with no evident autolysis or only slight putrefaction. Death was confirmed by complete cessation of opercular movement and absence of response to mechanical tactile stimulus. Tissue fixation was completed within 15 min after death confirmation for all D group individuals. Six surface sediment samples (0–5 cm) were collected from the same pond using a sediment sampler; each sample was homogenized individually in a sterile mortar prior to DNA extraction. These six sediment samples constituted the environmental microbial control group (S group). Sampling was carried out on an aseptic, clean workbench. The body surface of each fish was disinfected with 75% ethanol and placed on ice. Mid-intestinal tissue segments were collected and fully immersed in 4% paraformaldehyde solution for Hematoxylin and Eosin (H&E) staining and histological observation, while intestinal contents were obtained by gently squeezing the intestinal tract, and intestinal tissue samples were also retained. The intestinal contents were preserved in liquid nitrogen and subsequently stored at −80 °C.

The animals were treated according to the “Regulations on the Administration of Experimental Animals” formulated by the Department of Science and Technology of Guangdong Province. The experiments were conducted as approved by the Institutional Animal Care and Use Committee of Hanshan Normal University (No. 2026032401).

### 2.2. Histological Observation of Liver and Intestine

Liver and mid-intestinal tissues of all 18 fish individuals (6 per group) were fixed in 4% paraformaldehyde solution (Beijing Solarbio Science & Technology Co., Ltd. Beijing China). To standardize pathological evaluation, identical mid-intestinal segments, consistent transverse sectioning orientation, and unified 200× magnification were applied across all samples. Fixed tissue samples (approximately 1 cm^2^) were rinsed under running tap water for 4 h and subsequently dehydrated through a graded ethanol series (Xilong Scientific Co., Ltd., China) as follows: 60% ethanol for 1 h, 70% ethanol for 1 h, 80% ethanol for 30 min, 90% ethanol for 10 min, 95% ethanol for 5 min, and 100% ethanol for 5 min (twice). Tissues were then cleared in xylene (Xilong Scientific Co., Ltd., Shantou, China) for 5 min, infiltrated with paraffin at 65 °C for 30 min, and embedded in paraffin at 70 °C. Paraffin sections were cut and subjected to H&E staining. Briefly, sections were dewaxed in xylene for 10 min (twice), rehydrated through a descending ethanol gradient (100% ethanol 5 min, twice; 95% ethanol 5 min; 80% ethanol 5 min; 70% ethanol 5 min), and rinsed in deionized water. Nuclear staining was performed by immersing sections in Harris hematoxylin solution (Beijing Solarbio & Technology Co., Ltd., China) for 10 min, followed by rinsing with deionized water, brief differentiation in 1% acid alcohol, rinsing with distilled water, bluing in 0.6% ammonia water, and a final wash with tap water. Cytoplasmic staining was performed by immersing sections in eosin solution (Beijing Solarbio & Technology Co., Ltd., China) for 3 min. Stained sections were dehydrated through 95% ethanol for 5 min (twice) and 100% ethanol for 5 min (twice), cleared in xylene for 5 min (twice), air-dried briefly, and mounted with neutral mounting medium. Sections were examined under a light microscope at 200× magnification, and 3–5 non-overlapping fields of view were captured for each individual for pathological assessment. Objective diagnostic criteria were defined to distinguish genuine pathological vacuolation from artifacts introduced during sample processing: true pathological vacuoles were round, membrane-bound cytoplasmic cavities consistently observed across multiple fields, whereas irregular empty spaces only appearing in isolated fields were regarded as processing artifacts. All raw histological images will be submitted as Supplementary Materials upon manuscript acceptance.

### 2.3. DNA Extraction, PCR Amplification, and Sequencing

The total microbial DNA of intestinal contents or the composite sediment sample was extracted using the PowerFecal™ DNA Isolation Kit (MO BIO Laboratories, Carlsbad, CA, USA) according to the manufacturer’s protocols. Briefly, samples were subjected to bead-beating lysis, inhibitor removal, silica-column-based DNA binding, multiple washing purification steps, and final DNA elution. DNA integrity was verified by 1% agarose gel electrophoresis, while DNA concentration and purity were quantified using a NanoDrop 2000 spectrophotometer (Thermo Fisher Scientific, Wilmington, DE, USA). Acceptable A260/A280 ratios ranged from 1.8 to 2.0 for all samples. The hypervariable V3-V4 regions of the bacterial 16S rRNA genes were amplified by PCR using primers 341F (5′-CCTAYGGGRBGCASCAG-3′) and 806R (5′-GGACTACNNGGGTATCTAAT-3′). PCR reactions were performed in triplicate with a 20-μL mixture containing 4 μL of 5× FastPfu Buffer, 2 μL of 2.5 mM dNTPs, 5 μM of forward and reverse primers, 0.4 μL of FastPfu DNA Polymerase (TransStart, Beijing China), 10 ng of template DNA, and made up to the required volume with sterile water. The PCR conditions were as follows: 95 °C for 5 min, 27 cycles at 95 °C for 30 s, 55 °C for 30 s, and 72 °C for 45 s, with a final extension at 72 °C for 5 min. The PCR products were then extracted from the 1% agarose gels and purified with a DNA Gel Extraction Kit (Axygen, Hangzhou, China) according to the manufacturer’s instructions. The concentration of the purified DNA was quantified using a NanoDrop 2000 spectrophotometer. The genomic DNA of all these samples was submitted to a commercial sequencing provider (Novogene, Beijing, China) for 16S rRNA gene amplicon sequencing.

### 2.4. Bioinformatics Analysis of 16S rRNA Sequencing

After demultiplexing the reads according to sample-specific barcodes and trimming the primer sequences, raw data were obtained by merging the paired-end reads with FLASH (V1.2.11, http://ccb.jhu.edu/software/FLASH/, accessed on 5 January 2026) [37]. The merged sequences were subsequently processed with Fastp (V0.23.1) to remove low-quality data, generating a set of high-quality clean tags [38]. Chimeric sequences were detected and filtered by alignment against the Silva reference database, yielding an effective sequence dataset for downstream analysis [39] conducted using QIIME2, along with their taxonomic features [40]. Two separate feature tables were generated for different analytical workflows. Amplicon sequence variants (ASVs) were inferred via the DADA2 pipeline in QIIME2 and were exclusively used for FEAST source-tracking analysis. Sequences were further clustered into operational taxonomic units (OTUs) at 97% similarity for alpha diversity calculation, Venn diagram visualization, taxonomic-composition profiling, beta diversity clustering, LEfSe biomarker detection, and functional prediction. Alpha diversity metrics, including Chao1, ACE, and Goods coverage, were calculated in QIIME2. The Chao1 and ACE estimators were used to evaluate total species richness while accounting for rare taxa that may remain undetected due to limited sequencing depth, whereas Goods coverage was applied to validate the completeness of the observed diversity. Beta diversity analyses were performed by principal coordinate analysis (PCoA) and weighted pair-group method with arithmetic mean (WPGMA) clustering in QIIME2, enabling the visualization and comparison of microbial community structure across the experimental groups.

Differentially abundant microbial taxa among groups were identified by linear discriminant analysis effect size (LEfSe) with an LDA score threshold greater than 3.0. The statistical assessment of differences at the phylum and genus levels was conducted in the R software environment, applying the Kruskal–Wallis H-test followed by Dunn’s post-hoc multiple-comparison test with a significance cutoff of *p* < 0.05. Additionally, the taxonomic annotation profiles were mapped to functional reference databases, and the metabolic functional potential of the microbial communities was predicted using PICRUSt2 (version 2.4.1).

### 2.5. FEAST Analysis Based on the 16S rRNA Genes

The Fast Expectation-Maximization Microbial Source Tracking (FEAST) R package for microbial source tracking was used for this study. In the analysis, sediment samples (S group) were used as known sources, and various gut samples healthy (H), M, and D groups) were treated as sinks. The input data consisted of an ASV abundance matrix and a metadata table with sample group information labeled as ‘Source’ or ‘Sink’. The EM algorithm was run for 1000 iterations, simultaneously estimating the ‘unknown source’ proportion to account for microbial groups that cannot be attributed to known sources [41,42].

### 2.6. Data Analysis

All statistical analyses were performed in R (v4.2.1) and SPSS 20.0 with significance threshold set at *p* < 0.05. Differences in alpha diversity indices, taxonomic relative abundances, and PICRUSt2-predicted functional profiles across groups were evaluated by the Kruskal–Wallis H-test with Dunn’s post-hoc multiple-comparison test. Multivariate community differences were assessed via PERMANOVA and ANOSIM (999 permutations) based on weighted UniFrac distances. LEfSe was performed with an LDA cutoff of 3.0 for biomarker identification. FEAST source-tracking was executed with 1000 EM iterations as described above.

The irrelevant calculation of Pearson’s correlation between microbiota-taxon abundance and transcript expression was removed, as no transcriptomic dataset was generated in this study.

## 3. Results

### 3.1. Gross Appearance and Intestinal Structure of Spiny Eels Under Different Physiological States

**Figure 1:**
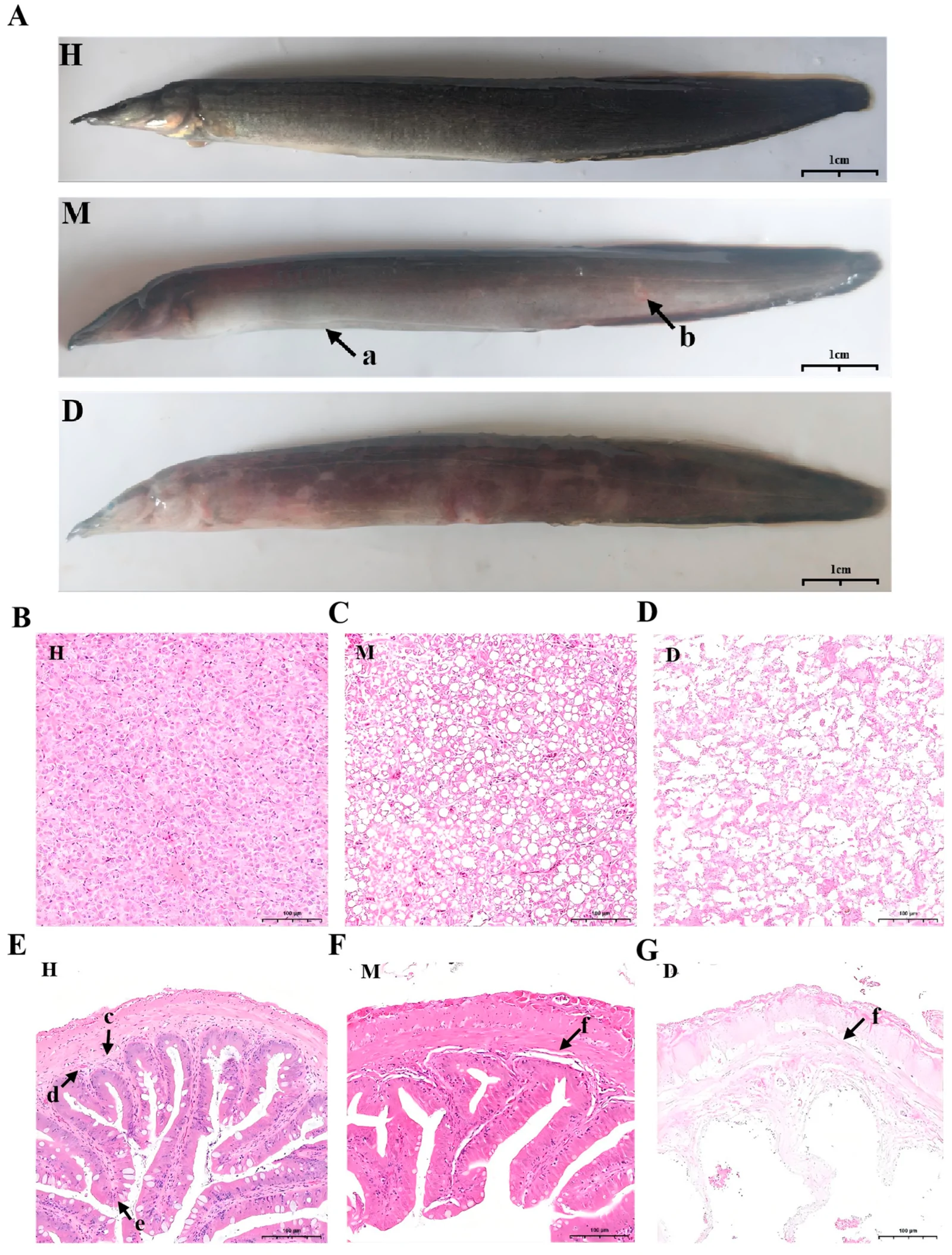
Clinical signs and histopathology of liver and intestinal tissues in spiny eels in different experimental groups: H: healthy group, M: moribund group, D: dead group. (**A**) Appearance of healthy (H), moribund (M), and dead (D) individuals. a, hepatic cyanosis; b, skin ulceration. Black arrows and indicate representative lesions: liver congestion spots (M, anterior body cavity) and caudal peduncle skin ulcers (M). Scale bars: (**A**) 1 cm (**B**–**D**) liver tissue sections (H&E staining). (**B**): normal hepatic cords and hepatocyte morphology. (**C**): extensive vacuolar degeneration and pyknosis, disorganized cords. (**D**): massive necrosis, parenchymal collapse. (**E**–**G**) Intestinal tissue sections (H&E staining). c, intestinal villus; d, mucosal epithelium; e, lamina propria; f, sloughed epithelial cells. (**E**): intact villi with clear layering. (**F**): villus atrophy, epithelial shedding (arrows), mucosal edema. (**G**): complete villus loss, only tissue debris. H, healthy; M, moribund; D, dead. Scale bars: (**B**–**G**) 100 µm.

The external morphology, liver, and intestinal histological features of healthy eels (H group) were compared with those of moribund (M group) and recently dead (D group) individuals to describe the pathological changes associated with physiological decline. Gross examination (Figure 1A) showed that H fish exhibited an intact body surface, abundant mucus, normal body coloration, and a well-rounded body without visible lesions. In contrast, M individuals displayed marked pathological signs, including reduced mucus secretion, body pallor, hepatic cyanosis accompanied by localized hyperemia, and partial skin ulceration, indicating severe surface damage and systemic functional deterioration at the moribund stage. Macroscopically, D group fish showed generalized pallor, loss of body sheen, widespread hyperemia, and edema. However, these external observations cannot rule out post-mortem autolysis occurring at the cellular level shortly after death.

Histological examination of the liver (Figure 1B–D) revealed intact hepatic architecture in the H group, with closely packed hepatocytes displaying regular morphology and no evidence of vacuolar degeneration or necrosis. In the M group, hepatocytes showed extensive genuine vacuolar degeneration (distinguished from processing artifacts based on multi-field observation), disordered hepatic cords, dilated sinusoids, and nuclear pyknosis and lysis, suggesting severe hepatic impairment. The hepatic parenchyma in the D group was completely disintegrated, with massive hepatocyte necrosis and only residual tissue debris remaining. Note that these hepatic alterations in D group individuals may partly reflect post-mortem autolysis and should not be simply interpreted as a linear continuation of ante-mortem disease progression.

Intestinal histology (Figure 1E–G) showed that H fish possessed intact intestinal architecture with well-organized villi and clearly discernible mucosal epithelium and muscularis layers. In the M group, intestinal villi were atrophied, epithelial cells were sloughed, and the mucosal layer was edematous, with partial detachment. The D group displayed a complete collapse of intestinal structure, with villi absent and only a fragmented tissue framework remaining.

### 3.2. 16S rRNA Gene Sequencing Analysis

To elucidate the changes in the gut microbiota of spiny eels along the healthy–moribund–dead continuum and their association with the sediment microbiome, 16S rRNA amplicon sequencing was performed on intestinal samples from H, M, and D groups, with pond sediment (S group) as an environmental reference. It should be noted that the S group represents a single pooled composite sediment sample without biological replicates; therefore, statistical comparisons between sediment and gut microbiota were not performed.

**Figure 2:**
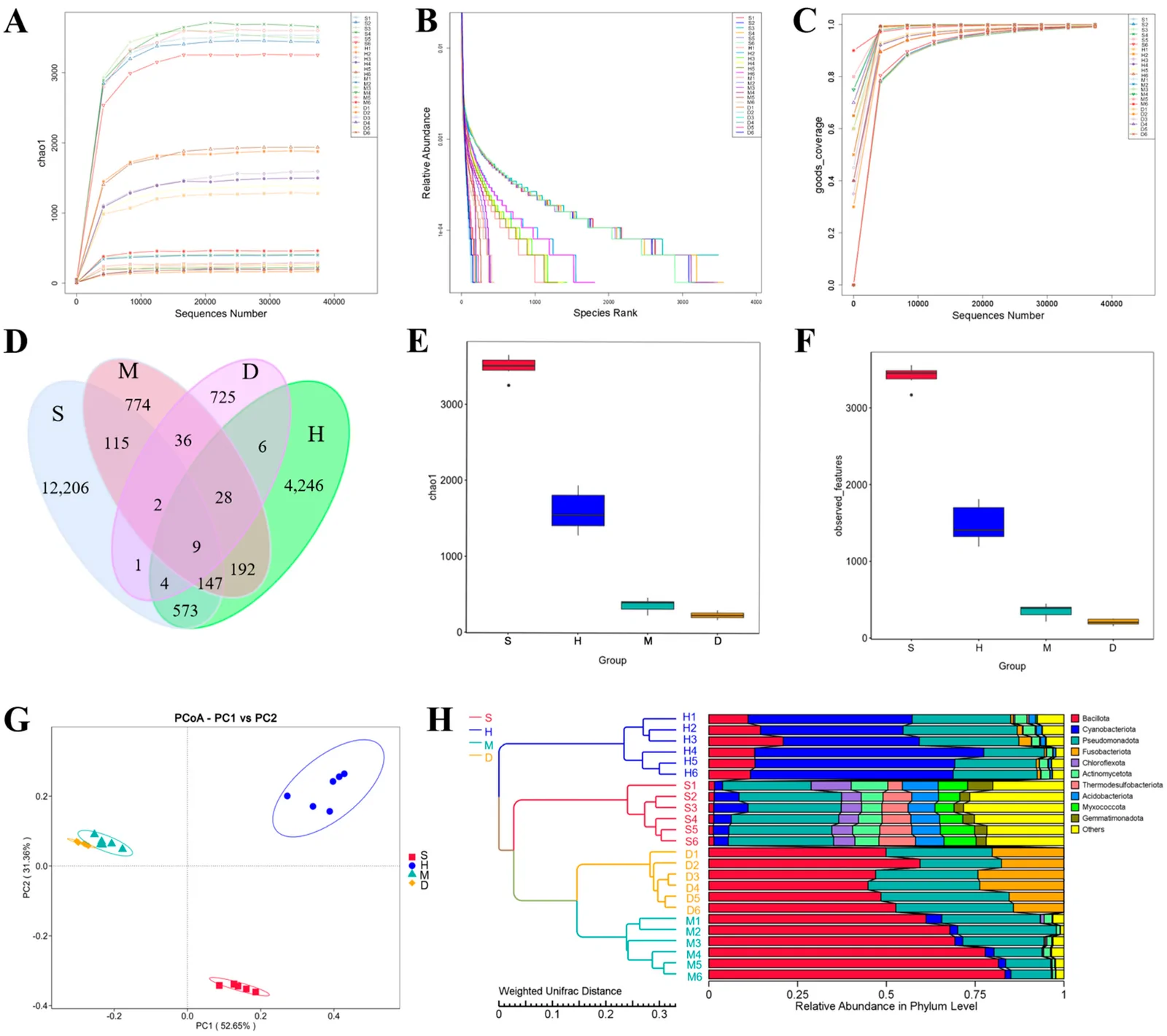
The 16S rRNA gene sequencing analysis of the gut microbiota of spiny eels in different experimental groups: S: sediment group, H: healthy group, M: moribund group, D: dead group, and the corresponding pond sediment. (**A**) Rarefaction curves of all samples; (**B**) Rank-abundance curves; (**C**) Goods coverage curves; (**D**) Venn diagram displaying the shared and unique OTUs among the sediment (S), healthy (H), moribund (M), and recently dead (D) groups. (**E**,**F**) Alpha diversity indices (Chao1 index and observed features). (**G**) Principal coordinate analysis (PCoA) plot based on weighted UniFrac distances. (**H**) Unweighted pair-group method with arithmetic mean (UPGMA) clustering dendrogram.

A total of 24 samples yielded 2,525,236 raw paired-end reads, and 2,498,963 tags were obtained after merging. The rarefaction curves reached a plateau (Figure 2A), indicating that the sequencing depth was sufficient to capture the microbial diversity in the samples. The rank-abundance curves demonstrated that all microbial species in the samples were effectively detected (Figure 2B). The Goods coverage values for all samples exceeded 99%, confirming the completeness of bacterial community detection (Figure 2C).

A total of 19,163 operational taxonomic units (OTUs) were identified across all samples. The Venn diagram (Figure 2D) showed that only nine OTUs were shared among the four groups. The S group (pond sediment samples) harbored the highest number of unique OTUs (12,206), which was markedly higher than that of intestinal samples. The number of unique OTUs in the H group (4246) was more than five times that in the M and D groups, indicating substantial compositional divergence between the sediment and gut microbiota as well as between healthy and pathologically impaired gut communities.

Alpha diversity analysis based on the Chao1 index and observed features (Figure 2E,F) revealed that the S group exhibited the highest microbial richness and diversity. Within the intestinal samples, the H group showed significantly higher diversity than the M and D groups, whereas no significant difference was observed between the M and D groups, suggesting that major diversity loss was already present at the moribund stage. Principal coordinate analysis (PCoA) based on weighted UniFrac distances and unweighted pair-group method with arithmetic mean (UPGMA) clustering (Figure 2G) demonstrated complete separation among the four groups, with the M and D groups displaying highly overlapping community structures.

PERMANOVA and ANOSIM tests further confirmed that the microbial community structures differed significantly among all groups (*p* < 0.01), with particularly pronounced differences between healthy individuals and moribund/dead individuals.

### 3.3. Alterations in Gut Microbial Community Composition

**Figure 3:**
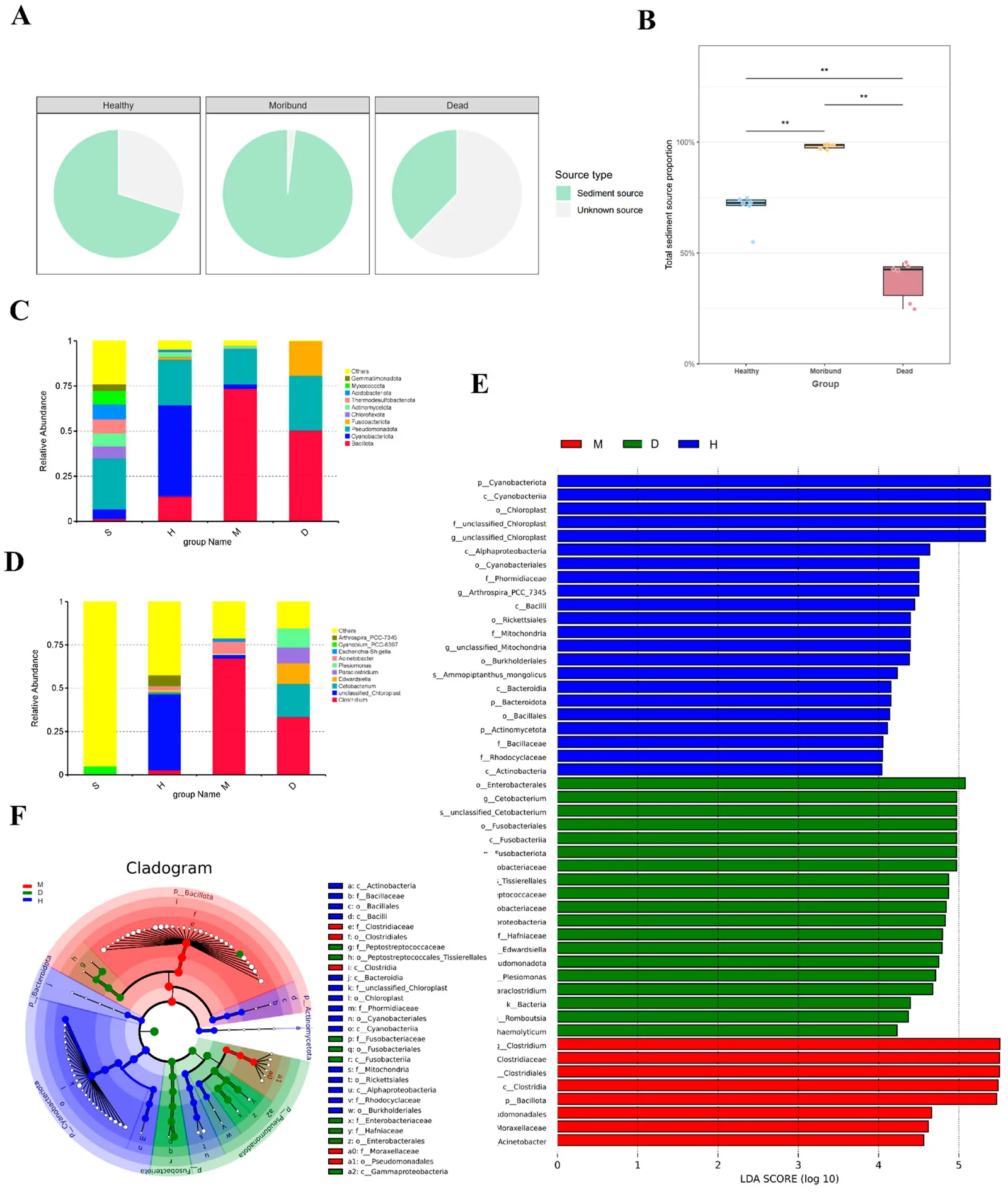
Microbial composition in different experimental groups (H: healthy group, M: moribund group, D: dead group) and the corresponding pond sediment. (**A**) Reconstruction of the intestinal microbiota composition of spiny eels in different experimental groups and the corresponding pond sediment. FEAST group pie chart showing the average proportion of sediment-derived and unknown sources in the gut microbiota of H, M, and D groups. The sediment source accounted for about 28% in the H group, while it was around 99% and 43% in the M and D groups, respectively. (**B**) Boxplot of sediment source proportions, showing the distribution of sediment-derived proportions in individual samples across groups and the significance of differences among groups (Kruskal–Wallis test, ** *p* < 0.01). (**C**) Top 10 bar chart of phylum-level relative abundance, showing the distribution changes of dominant phyla in each group. (**D**) Top 10 bar chart of genus-level relative abundance. (**E**) LEfSe analysis LDA score bar chart (LDA > 3), showing biomarkers with significantly different abundances among groups. (**F**) LEfSe cladogram, showing the phylogenetic distribution of differential species from phylum to genus levels.

To clarify the specific taxonomic changes in gut microbiota under the influence of natural environmental fluctuations in water sources and their potential sources, we used FEAST to perform conditional microbial source-attribution analysis and analyze community composition at the phylum and genus levels, screening for differentiating markers using LEfSe. Since only sediment was set as the known source in the FEAST model and other potential microbial reservoirs were not included, all FEAST outputs represent conditional estimates rather than direct proof of sediment-to-gut bacterial invasion. FEAST source tracking (Figure 3A,B) showed that sediment accounted for only about 28% on average in the gut microbiota of the H group, indicating that the gut microbiota of healthy individuals mainly relies on host endogenous maintenance. In contrast, sediment accounted for about 99% and 44% in the M and D groups, respectively, showing a highly significant difference compared with the H group (Kruskal–Wallis test, *p* < 0.01). This suggests that the host’s selective filtering function for gut microbes has severely collapsed during the moribund stage, and the gut microbiota was almost entirely replaced by microbes from environmental sediments.

At the phylum level (Figure 3C), Proteobacteria dominated the gut of eels in the H group. In the M and D groups, the relative abundance of Proteobacteria sharply decreased, while Firmicutes and Fusobacteriota became dominant, indicating a fundamental ecological niche shift at the phylum level. At the genus level (Figure 3D), *Cetobacterium* was the core dominant genus in the H group, making up about 40% of the relative abundance. In the M and D groups, *Cetobacterium* almost completely disappeared (red dashed box); potentially opportunistic taxa such as *Fusobacterium* and *Clostridiaceae* showed markedly increased relative abundance (blue dashed box), becoming the dominant groups. We note that genus-level 16S rRNA data cannot confirm true pathogenicity; strain-level or metagenomic evidence would be required for such verification. This inverse pattern reveals the key taxonomic differences between healthy and moribund individuals.

LEfSe analysis (LDA > 3.0) (Figure 3E,F) further identified differential markers for each group. The H group was significantly enriched with Actinobacteria, Proteobacteria, and *Cetobacterium*. M and D groups were both enriched with Firmicutes, Fusobacteria, and the families Fusobacteriaceae and Clostridiaceae. The two groups showed a high overlap in marker lineages, further supporting that the gut microbiota in the moribund stage has shifted to an irreversible pathological steady state. Bear in mind that microbiota profiles of D group fish may be confounded by post-mortem microbial succession.

### 3.4. Functional Alterations of the Gut Microbiota

**Figure 4:**
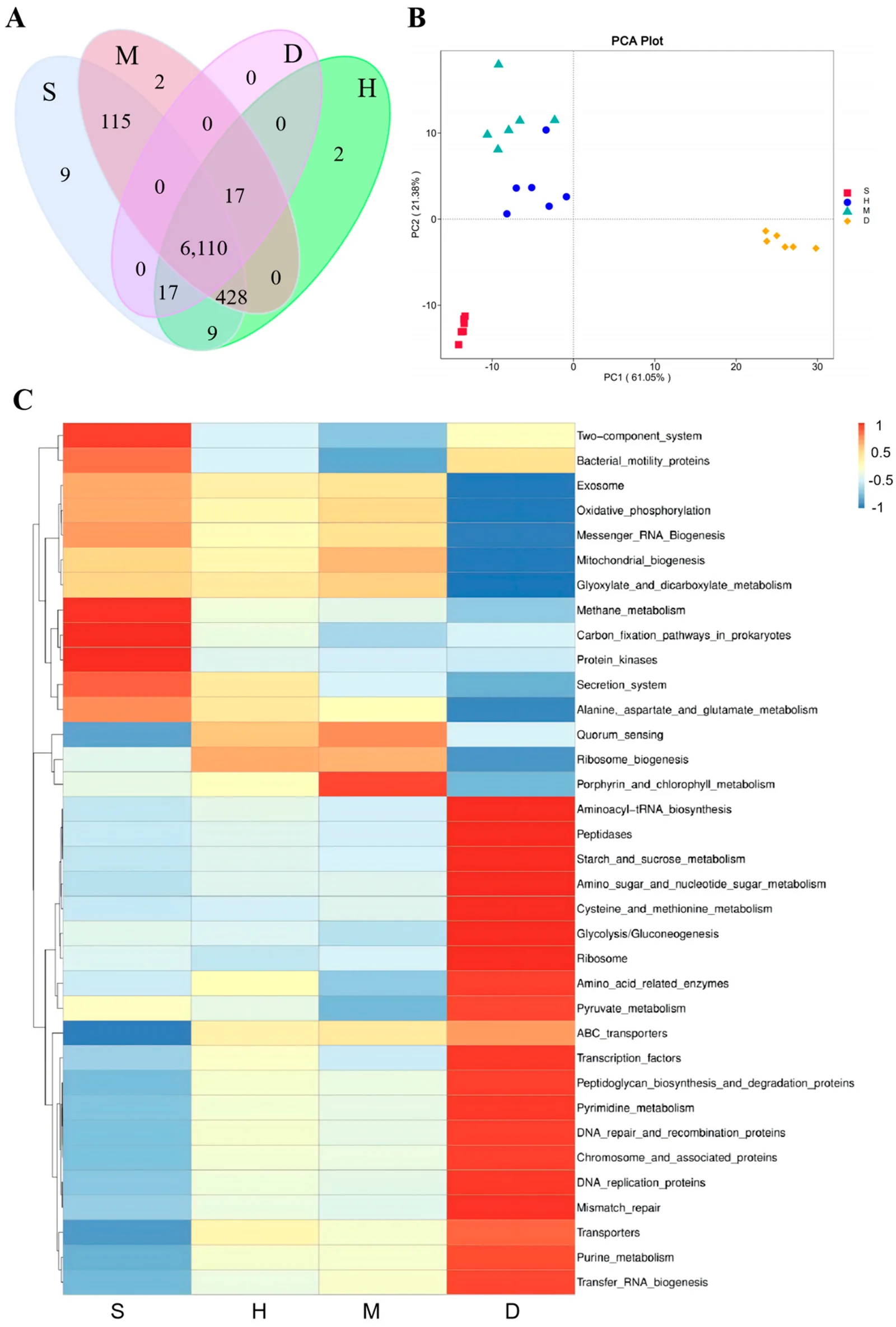
Functional changes in the gut microbiota of spiny eels in different experimental groups (H: healthy group; M: moribund group; D: dead group) and the corresponding pond sediment. (**A**) Venn diagram of shared and unique functional pathways among the four groups. (**B**) Principal component analysis (PCA) of microbial functional profiles. (**C**) Heatmap of KEGG functional abundance at level 3.

To assess the predicted functional potential associated with gut microbiota community structure reconstruction, we performed a functional prediction analysis based on 16S rRNA gene data. The Venn diagram of functional pathways (Figure 4A) shows that only 6110 KEGG Ortholog (KO) entries were shared among the four groups, indicating significant functional differences between physiological states and between gut and sediment environments. The PCA analysis (Figure 4B) shows that the microbial functions in the H group and the M/D group were completely separate, while the M and D groups have a high degree of overlap. PC1 and PC2 explain 61.05% and 21.38% of the total variance, respectively, suggesting that gut microbiota have undergone a global functional reconstruction under different physiological states, and this functional shift is essentially established by the terminal stage.

Sediment (S) samples are clearly separated from all gut samples in the functional space, further indicating that the functional characteristics of the gut microbiota are not derived from the environment but are shaped by host–microbe symbiosis. The KEGG functional heatmap (Figure 4C) shows that the H group is characterized by a high abundance of pathways related to oxidative phosphorylation and mitochondrial biosynthesis, representing symbiotic energy metabolism pathways. In contrast, these functional categories exhibited lower predicted relative abundance in the M and D groups; KO entries associated with bacterial proliferation and tissue degradation (such as peptidases, glycolysis, and peptidoglycan biosynthesis) are significantly enriched. The S group is enriched with environmental survival functions such as the two-component system and bacterial motility proteins, clearly distinct from the gut functional spectrum. Importantly, these outputs represent PICRUSt2 predicted functional potentials and do not reflect actual enzyme activities or in vivo gene expression; predicted enrichment of peptidase-related functions cannot directly prove that bacterial enzymes caused the observed histological tissue damage.

## 4. Discussion

This study describes associations between gut microbiota remodeling and host physiological decline in spiny eels, and evaluates potential links with the sediment microbiota. It should be noted that chronic sediment stress is treated as a plausible field hypothesis rather than a proven conclusion in the present observational cross-sectional study. Histopathology revealed that by the moribund stage, the intestinal epithelium was severely compromised (villus atrophy, epithelial shedding, and mucosal edema), and the liver showed extensive vacuolar degeneration and pyknosis. Host tissue damage and microbial dysbiosis are strongly associated, whereby tissue impairment may create a permissive environment for potentially opportunistic taxa. Alpha diversity declined sharply from healthy to moribund individuals, with no further significant change after death, indicating that major richness loss was already evident at the moribund stage, consistent with stressed fish [43,44]. *Cetobacterium*, a keystone vitamin B12 producer and mucosal regulator, nearly disappeared [45,46]. It ferments carbohydrates to short-chain fatty acids that fuel colonocytes, strengthen tight junctions, and exert anti-inflammatory effects [47,48]. Its decline weakens epithelial integrity and immunity, accelerating detachment and edema [49]. Firmicutes and Fusobacteriota rose while Proteobacteria fell, a characteristic dysbiosis. Though Proteobacteria dominance indicates instability in mammals, in many freshwater fish it typifies a healthy microbiota [50,51]. The shift to Firmicutes under ammonia and hypoxia stress links to intestinal inflammation and pathogen overgrowth [52,53], reflecting both chemical toxicity and altered host immunity.

The moribund stage is the critical tipping point. PCoA and UPGMA showed that moribund and dead communities overlap but are distinct from healthy individuals, indicating that an altered pathological microbial state is present at the moribund stage [54,55]. LEfSe identified *Cetobacterium* as a healthy biomarker, whereas Firmicutes, including Clostridiaceae, and Fusobacteriaceae characterized the moribund state. Clostridiaceae and *Fusobacterium* are proteolytic, mucin-degrading bacteria that thrive when the mucosal barrier is disrupted and are implicated in fish enteritis [56,57]. Enrichment of these potentially opportunistic taxa in moribund fish and their persistence in recently dead individuals suggests that intervention strategies should target earlier stages of physiological decline. In addition, the *Cetobacterium*/Firmicutes relative abundance ratio may have potential as an early warning monitoring indicator, though this requires further validation in larger-scale sampling. Despite high sediment diversity (>12,000 OTUs), gut–sediment overlap was minimal (nine shared OTUs), with complete ordination separation, demonstrating that gut microbiota is shaped by host-specific filters (pH, bile acids, oxygen, and mucosal immunity) rather than passive ingestion [58]. FEAST quantified this: about 28% of healthy gut microbes came from sediment, indicating host-driven selection, similar to other fish where genetics and immunity maintain a core community [59,60].

Nevertheless, we found that taxa blooming in moribund guts (Fusobacteriaceae, Clostridiaceae) were detectable in the sediment at low abundance, suggesting that sediment may act as a potential microbial reservoir that could influence gut microbiota composition. We note that our study cannot directly verify the causal role of chronic sediment stress; reports linking degraded-sediment conditions (ammonia, nitrite, hypoxia) to gill–intestinal barrier damage and microbial entry represent findings from independent published literature [61]. The FEAST results directly provide evidence for this invasion process, with the proportion of sediment sources soaring to about 99% in moribund individuals and reaching 44% in dead ones, showing that the host’s selective filtering had already seriously collapsed even before death. As a result, the gut of moribund individuals becomes a nutrient-rich niche, where fast-growing anaerobes outcompete the stressed symbionts. This process may be further amplified by a positive feedback loop in which the moribund host releases gut bacteria into the surrounding sediment through excretion and decomposition, increasing the environmental pathogen load for the remaining population [62]. Our inclusion of freshly dead individuals supports this, yet the exact microbial exchange dynamics remain to be confirmed in further controlled studies. However, the exact microbial exchange dynamics remain to be confirmed by further controlled studies.

Functional predictions derived from PICRUSt2 suggested a shift from oxidative phosphorylation and mitochondrial biogenesis in the healthy gut to peptidoglycan biosynthesis, glycolysis, and peptidases in the moribund and dead groups. The upregulation of peptidoglycan biosynthesis may indicate actively dividing bacterial populations, consistent with an opportunistic bloom, while increased peptidase activity suggests degradation of host extracellular matrix and mucosal proteins, correlating with the histological damage observed [63]. The results indicate a similarity in the functional characteristics of the gut microbiota between the moribund and dead groups. This functional reprogramming may contribute to host tissue breakdown, as bacterial enzymes penetrate deeper into the compromised mucosa. Although PICRUSt2 predictions are limited by the resolution of 16S rRNA gene amplicon data and cannot directly quantify enzymatic activities, the consistent pattern across multiple pathway categories provides strong evidence for a genuine ecological transition that needs to be confirmed by metagenomic and metabolomic methods [64].

Collectively, these findings provide practical hints for spiny eel aquaculture management: microbial biomarkers (*Cetobacterium*, Clostridiaceae) and the *Cetobacterium*/Firmicutes ratio may serve as early indicators of physiological decline, and benthic habitat management merits attention for this benthic fish. It is worth noting the technical limitations of this field-based observational study. Our findings reflect biological associations instead of definitive causal relationships. Limitations include pooled composite sediment samples lacking biological replicates, FEAST using only sediment as the known source, insufficient strain-level resolution from 16S rRNA data, absence of sediment geochemical measurements, and potential post-mortem microbial interference in D group samples. Further multi-pond sampling and controlled challenge experiments will help validate these observed patterns and test relevant causal hypotheses.

## 5. Conclusions

This study reveals a gradual decline in the gut microbiota of spiny eel along a natural mortality gradient under the influence of chronic sediment stress. The results indicate that disruption of the host barrier and dysbiosis are interconnected, with sediment acting as a potential reservoir of pathogens. Our findings provide a meaningful framework for explaining the gradual disruption of the gut microbiota in the benthic fish undergoing chronic sediment-associated stress, and open new avenues for developing applied strategies for host health monitoring and aquaculture management. Future repeated multi-pond and seasonal surveys could further evaluate the applicability of the identified bioindicators and functional profiles in diverse environmental contexts.

## Data Availability

The data presented in this study are available on request from the corresponding author (M.Z.).

## Supplementary Materials

The following supporting information can be downloaded at: https://www.mdpi.com/article/10.3390/ani16213350/s1.

## Abbreviations

The following abbreviations are used in this manuscript:

H	Healthy
M	moribund group
D	Dead group
S	Sediment
DNA	Deoxyribonucleic acid
PCR	Polymerase chain reaction
rRNA	Ribosomal ribonucleic acid
OTUs	Operational taxonomic units
PCoA	Principal coordinate analysis
UPGMA	Unweighted pair-group method with arithmetic mean
FEAST	Fast Expectation-Maximization Microbial Source Tracking
ANOVA	Analysis of variance
PERMANOVA	Permutation Multivariate Analysis of Variance
ANOSIM	Analysis of Similarities
