# Supplementary material for: Gut Microbiota Alterations and Intestinal Pathology in Healthy, Moribund, and Recently Dead Mastacembelus Armatus from a Pond Aquaculture System
Source: Animals (Basel). 2026 Oct 23;16(21):3350. doi: 10.3390/ani16213350 (PMC13761855; doi:10.3390/ani16213350)
Supplement: Supplementary file 1 [file animals-16-03350-s001.zip › Graph/Replace images in Figure 2 and Figure 4/Fig2.pdf]

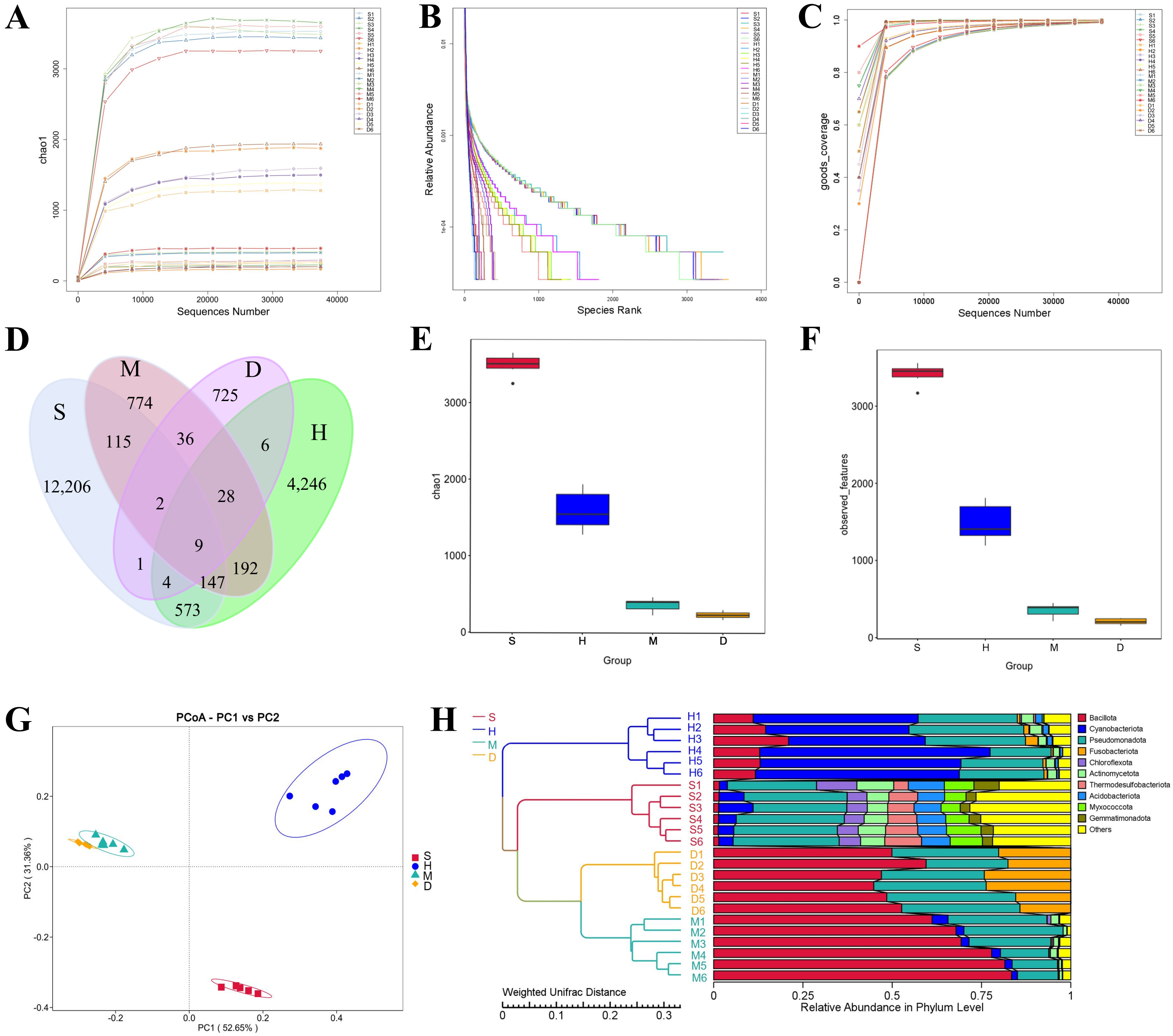

A
chao1
Sequences Number
B
Relative Abundance
Species Rank
C
goods_coverage
Sequences Number
D
S
M
D
H
774
725
115
36
6
12,206
4,246
2
28
9
1
192
4
147
573
E
chao1
Group
S
H
M
D
F
observed_features
Group
G
PCoA - PC1 vs PC2
PC1 ( 52.65% )
PC2 ( 31.36% )
H
Weighted Unifrac Distance
Relative Abundance in Phylum Level
Bacillota
Cyanobacteriota
Pseudomonadota
Fusobacteriota
Chloroflexota
Actinomycetota
Thermodesulfobacteriota
Acidobacteriota
Myxococcota
Gemmatimonadota
Others
