# Supplementary material for: Gut Microbiota Alterations and Intestinal Pathology in Healthy, Moribund, and Recently Dead Mastacembelus Armatus from a Pond Aquaculture System
Source: Animals (Basel). 2026 Oct 23;16(21):3350. doi: 10.3390/ani16213350 (PMC13761855; doi:10.3390/ani16213350)
Supplement: Supplementary file 1 [file animals-16-03350-s001.zip › Graph/Replace images in Figure 2 and Figure 4/Fig2E.pdf]

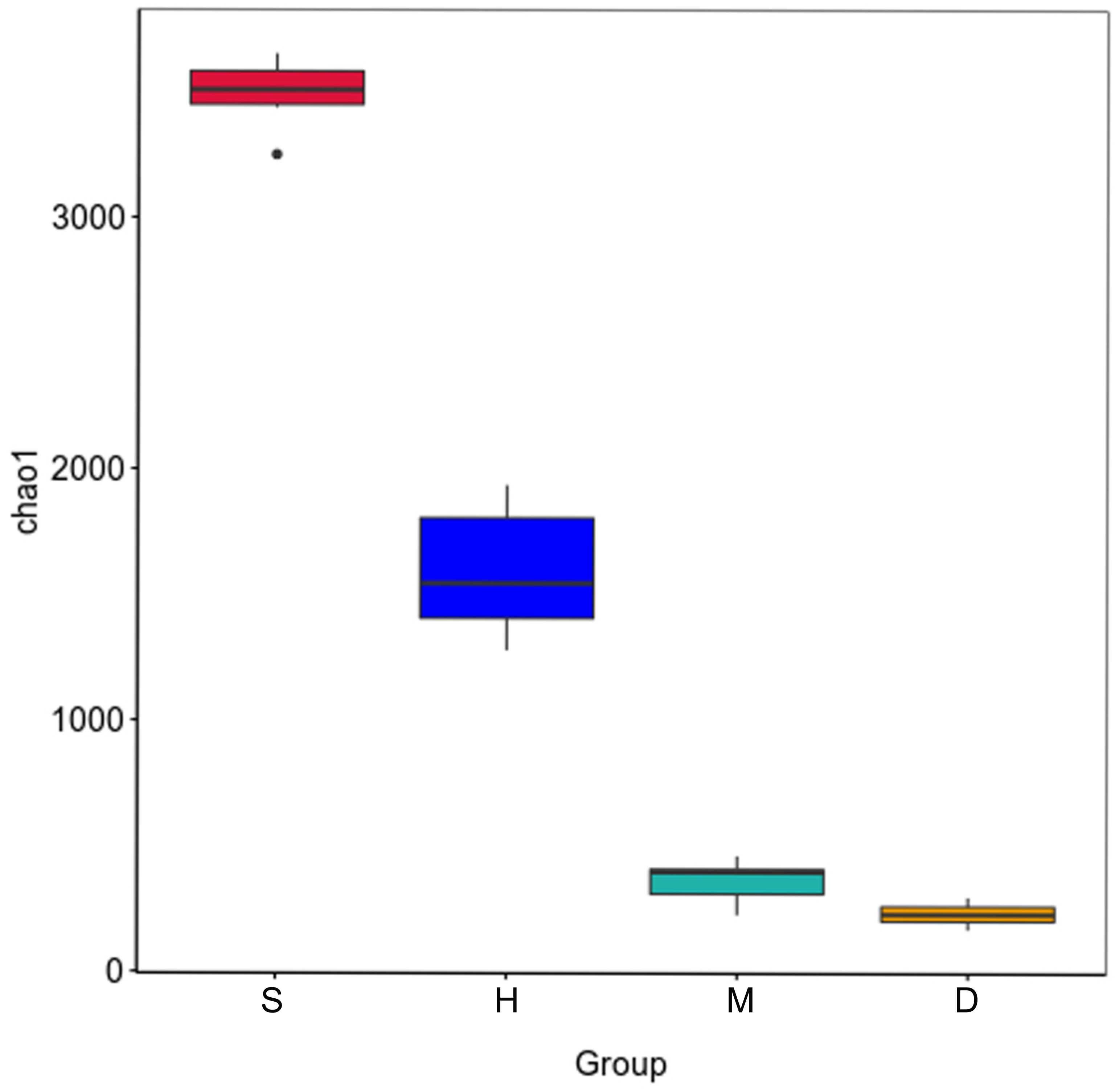
chao1
3000
2000
1000
0
S
H
M
D
Group
