# Supplementary material for: Gut Microbiota Alterations and Intestinal Pathology in Healthy, Moribund, and Recently Dead Mastacembelus Armatus from a Pond Aquaculture System
Source: Animals (Basel). 2026 Oct 23;16(21):3350. doi: 10.3390/ani16213350 (PMC13761855; doi:10.3390/ani16213350)
Supplement: Supplementary file 1 [file animals-16-03350-s001.zip › Graph/Replace images in Figure 2 and Figure 4/Fig2H.pdf]

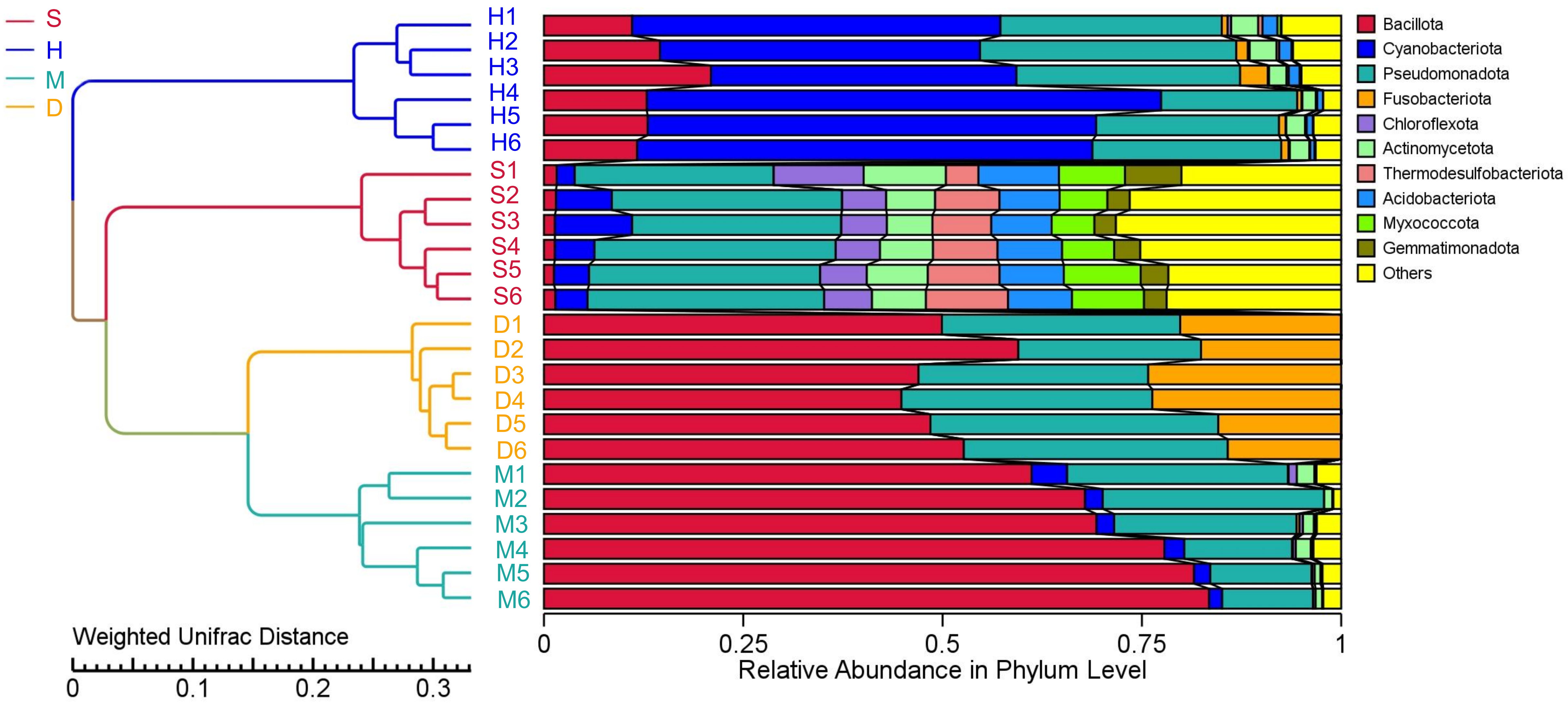
S
H
M
D
H1
H2
H3
H4
H5
H6
S1
S2
S3
S4
S5
S6
D1
D2
D3
D4
D5
D6
M1
M2
M3
M4
M5
M6
Weighted Unifrac Distance
0
0.1
0.2
0.3
0
0.25
0.5
0.75
1
Relative Abundance in Phylum Level
Bacillota
Cyanobacteriota
Pseudomonadota
Fusobacteriota
Chloroflexota
Actinomycetota
Thermodesulfobacteriota
Acidobacteriota
Myxococcota
Gemmatimonadota
Others
