# Supplementary material for: Gut Microbiota Alterations and Intestinal Pathology in Healthy, Moribund, and Recently Dead Mastacembelus Armatus from a Pond Aquaculture System
Source: Animals (Basel). 2026 Oct 23;16(21):3350. doi: 10.3390/ani16213350 (PMC13761855; doi:10.3390/ani16213350)
Supplement: Supplementary file 1 [file animals-16-03350-s001.zip › Graph/Replace images in Figure 2 and Figure 4/Fig4C.pdf]

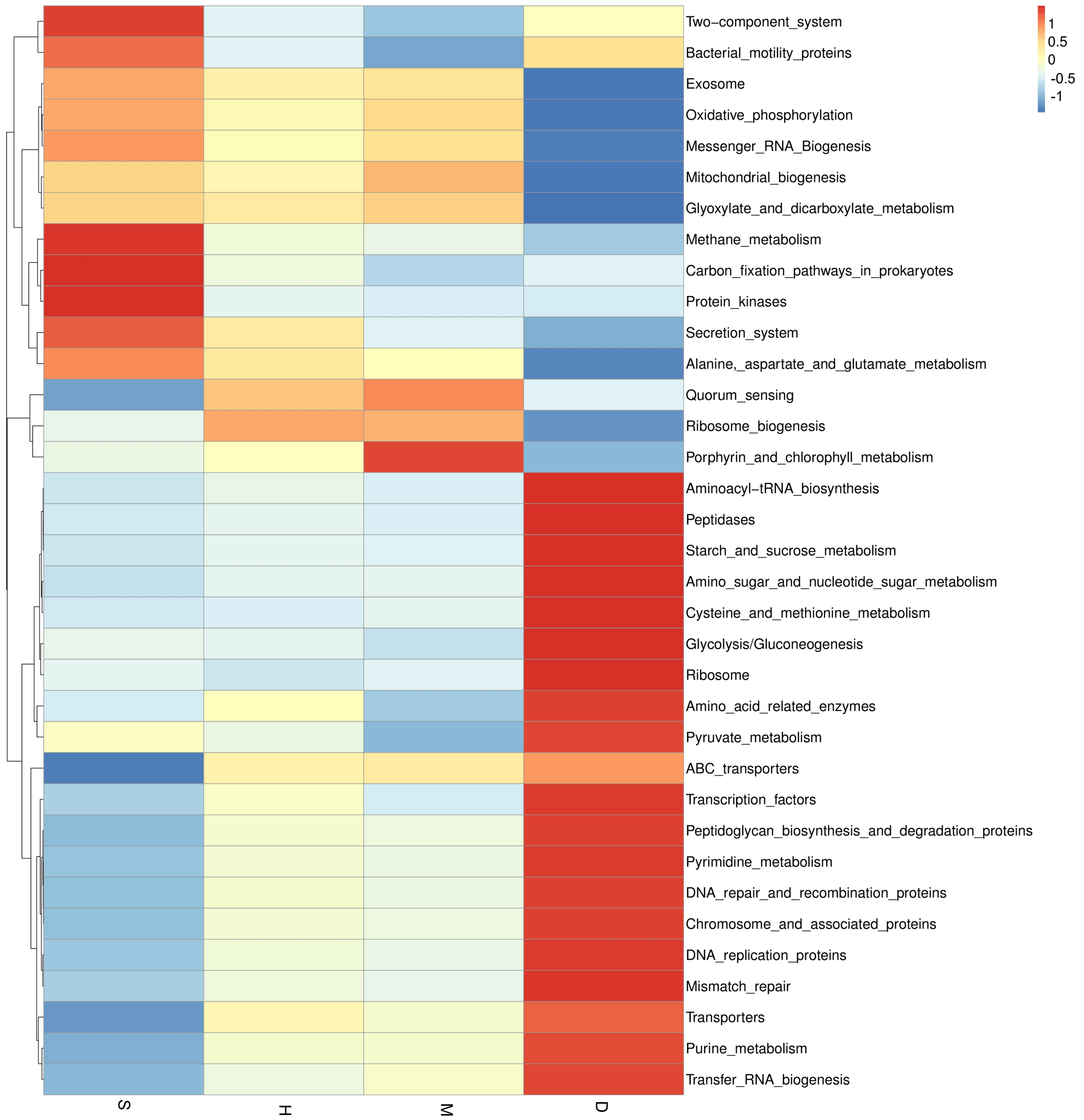
Two−component_system
Bacterial_motility_proteins
Exosome
Oxidative_phosphorylation
Messenger_RNA_Biogenesis
Mitochondrial_biogenesis
Glyoxylate_and_dicarboxylate_metabolism
Methane_metabolism
Carbon_fixation_pathways_in_prokaryotes
Protein_kinases
Secretion_system
Alanine,_aspartate_and_glutamate_metabolism
Quorum_sensing
Ribosome_biogenesis
Porphyrin_and_chlorophyll_metabolism
Aminoacyl−tRNA_biosynthesis
Peptidases
Starch_and_sucrose_metabolism
Amino_sugar_and_nucleotide_sugar_metabolism
Cysteine_and_methionine_metabolism
Glycolysis/Gluconeogenesis
Ribosome
Amino_acid_related_enzymes
Pyruvate_metabolism
ABC_transporters
Transcription_factors
Peptidoglycan_biosynthesis_and_degradation_proteins
Pyrimidine_metabolism
DNA_repair_and_recombination_proteins
Chromosome_and_associated_proteins
DNA_replication_proteins
Mismatch_repair
Transporters
Purine_metabolism
Transfer_RNA_biogenesis
S
H
M
D
1
0.5
0
-0.5
-1
